# Heparin-induced Thrombocytopenia in Acute Coronary Syndrome

**DOI:** 10.7759/cureus.4359

**Published:** 2019-04-01

**Authors:** Naresh Kumar, Suresh Kumar, Anil Kumar, Tariq Shakoor, Amber Rizwan

**Affiliations:** 1 Cardiology, Shalamar Hospital, Lahore, PAK; 2 Internal Medicine, Bolan Medical College, Quetta, PAK; 3 Cardiology, Punjab Institute of Cardiology, Lahore, PAK; 4 Family Medicine, Civil Hospital, Karachi, PAK

**Keywords:** acute coronary syndrome, heparin-induced thrombocytopenia (hit), heparin, myocardial infarction

## Abstract

Introduction

Heparin-induced thrombocytopenia (HIT) is by far the most relevant pathological association of it encountered by clinicians. It is an immune-mediated phenomenon caused by antibodies directed against complexes of heparin molecules and platelet factor 4 (PF4). HIT is a considerable side effect in patients of acute coronary syndromes (ACS). Its prevalence and associated outcomes in ACS patients have not been studied sufficiently except for clinical trials. The objective of this study was to assess the frequency of HIT in patients presenting with ACS.

Methods

This was an observational study with 272 patients between 40 and 70 years of either gender presenting with ACS within 24 hours of the first appearance of symptoms. Blood samples for baseline platelet count were taken before heparin therapy. Then, patients were subsequently administered low molecular weight heparin 5000 units stat, followed by 12 units/kg/hr for 72 hours of intravenous infusion. Blood samples were repeated for platelet count on Day 5. Thrombocytopenia was defined as per the recommendation of American College of Cardiology as " ≥50% decline in platelets (below 150 x 10^9^/L in most patients), which may occur immediately following heparin exposure (rapid presentation) or up to three weeks following exposure (delayed presentation)." Reports were assessed for the level of platelets. Data were entered and analyzed using SPSS version 22 (IBMCorp, Armonk, NY, US).

Results

The incidence of HIT was observed in 9.56% (n=26). In the HIT group, the mean platelet count on Day 5 was 109.81 ± 78.06 x 10^9^/L. The incidence of HIT in ST-segment elevation myocardial infarction (STEMI) and non-ST-segment elevation myocardial infarction (NSTEMI) were equal but higher than that in unstable angina (UA) (p-value=0.01). The incidence of HIT was also significantly higher in the group that presented late to the hospital (after 12 hours of symptom onset) (p-value=0.001).

Conclusion

The risk of HIT is more prominent in patients with myocardial infarction and in those who have a duration of symptoms more than 12 hours at the time of hospital presentation. Cardiologists and specialists of internal medicine have to take precautions while administrating heparin therapy in these high-risk patients, to avoid any complications.

## Introduction

Acute coronary syndrome (ACS), by definition, is the pathological rupture of an atherosclerotic plaque within the coronary artery endothelium, resulting in partial or complete thrombosis of the artery and subsequent infarction of the cardiac muscle. On electrocardiography (ECG), it may present as ST-segment elevation (STEMI), non-ST-segment elevation myocardial infarction (NSTEMI), or unstable angina (UA) [1-2].

The management of these patients consists of invasive therapy, such as percutaneous coronary intervention (PCI) and/or aggressive medical therapy in low-risk patients [1,3]. In ACS, conventionally, anti-platelets and anticoagulation are the mainstays of therapy for invasive as well as conservative strategy, according to American Heart Association guidelines [3].

Heparin is the most commonly used anticoagulant in patients presenting with ACS, which is associated with reduced risk of myocardial infarction and death in patients with UA [4].^ ^Understandably, despite many benefits, heparin has a considerable side-effect profile as well. One of these side effects is its immune reactivity against native platelets, causing heparin-induced thrombocytopenia (HIT) [5]. There are two types of HIT: when there is thrombocytopenia without thrombosis and when thrombocytopenia is associated with thrombosis. The pathogenesis of HIT involves the formation of antibodies against heparin-platelet factor 4 (PF4) complexes [5-6]. These antibodies, through their pro-coagulant effect, cause microvascular and macrovascular thrombosis, resulting in devastating complications, including subcutaneous bleeding, skin necrosis, venous limb gangrene, and adrenal vein thrombosis, causing hypotension. All these complications may succumb the patient to death if not treated on time, with appropriate measures [6]. The typical presentation of HIT, which is thrombocytopenia with or without vascular thrombosis, which is defined by American College of Cardiology (ACC) as a fall in platelet count below 150 × 109/L [7]. Early identification of HIT is essential for the appropriate clinical management of affected patient [5].The percentage of patients suffering from HIT according to studies varies from as low as 0.3% to as high as 13%[8-9].

The purpose of this study is to assess the frequency of HIT in patients presenting with ACS. In HIT, there is a worsening of existing thrombosis or the occurrence of thrombosis in new vascular territories. At times, heparin is continued or increased in dosage to counter these thrombotic events while treatment is actually to stop heparin. This peculiar nature requires the cardiologist to be extra vigilant when administering heparin to a patient in the setting of ACS. Because it is a common and usually underdiagnosed entity, it proves to be fatal if it goes unrecognized and mismanaged. Although there is one local study on HIT in patients undergoing cardiac bypass surgeries [7], there is a lack of studies on HIT in patients presenting with ACS conducted in a national setting. Through this study, we have made an attempt to determine the pattern of HIT.

## Materials and methods

This study was conducted at the Shalamar Hospital, Lahore, from June 2017-18. Inclusion criteria included patients aged between 40 and 70 years presenting with ACS within 24 hours of onset of symptoms. Exclusion criteria included patients with idiopathic thrombocytopenic purpura (ITP), aplastic anemia, myeloproliferative disorders, and pre-existing thrombocytopenia. Thrombocytopenia was assessed through medical records and by complete blood count (CBC), having isolated thrombocytopenia (platelet <150 x109/L) before heparin therapy. Patients who were lost to follow-up - either transferred to another hospital or died during the hospital stay - were also excluded. During the study period, 393 patients with ACS were admitted in the cardiology unit. Forty-four patients already had a duration of symptoms more than 24 hours when presenting (either delayed presentation or referred from another hospital). Another 48 patients were excluded due to either of the above-mentioned comorbidities. Out of the remaining 301 patients, 19 died during the hospital stay and 10 either left against medical advice or were transferred to another hospital by the family. Hence, 272 patients completed this study.

The baseline platelet count before the initiation of heparin therapy was recorded. Low molecular weight heparin 5000 units stat and then 12 units/ kg/ hr, for 72 hours, intravenous was initiated. On Day 5, blood samples were repeated for platelet count. Thrombocytopenia was labeled as platelet <150 x109/L, as recommended by ACC [7].

Data were analyzed using SPSS for Windows version 22.0 (IBM Corp, Armonk, NY, USA). Quantitative variables like age and platelet count were presented with mean and standard deviation (SD). Gender and heparin-induced thrombocytopenia were presented with frequency and percentage. Data were stratified for gender (male/female), age (40-70 years), type of ACS, time of presentation of the ACS, and platelet count at presentation. Stratified groups were compared by using the chi-square test, taking p-value <0.05 as significant.

## Results

There were 134 (49.3%) men and 138 (50.7%) women. Their mean age was 58.49 ± 5.87 years. Unstable angina was the most common form (n=119; 43.75%) and NSTEMI was the least common presentation (n=52; 19.1%). Overall, the incidence of heparin-induced thrombocytopenia was 9.5% (n=26). The demographic and clinical characteristics of the patients are shown in Table 1.

**Table 1:** Demographic and clinical characteristics of the patients (n=272) ACS: Acute coronary syndrome; MI: Myocardial infarction; ST: ST segment; SD: Standard deviation

Patient characteristics	Frequency n (%)
Gender	
Male	134 (49.3%)
Female	138 (50.7%)
Age in years	
40-55	82 (30.15%)
56-70	190 (69.85%)
Mean ± SD	58.49 ± 5.87
Co-morbidity	
Diabetes mellitus	106 (38.9)
Hypertension	89 (32.7%)
Previous history of ACS	114 (41.9%)
Type of ACS	
Unstable angina	119 (43.75%)
Non-ST elevation MI	52 (19.1%)
ST elevation MI	101 (37.1%)
Incidence of heparin-induced thrombocytopenia	26 (9.5%)

The mean platelet count of the patients on Day 0 was 281.39 ± 41.81 (x 109/L), which reduced to 232.74 ± 52.27 on Day 5 (x 109/L). In the HIT group, the mean platelet count on Day 5 was 109.81 ± 78.06 x 109/L. The demographic and clinical characteristics of patients diagnosed with HIT as compared to those who did not develop HIT are shown in Table 2, which shows that the HIT incidence in STEMI and NSTEMI were equal but higher than that in UA. The incidence of HIT was also significantly associated with the delayed presentation of ACS (Table 2).

**Table 2:** Characteristics of the patients diagnosed with heparin-induced thrombocytopenia (n=272) ACS: acute coronary syndrome; MI: myocardial infarction

	Heparin-Induced Thrombocytopenia		p-value
	Yes (n=26)	No (n=246)	
Gender			
Male	13 (9.7%)	121 (90.3%)	0.92
Female	13 (9.4%)	125 (90.6%)	
Age in years			
40-55	7 (8.5%)	75 (91.5%)	0.70
56-70	19 (10.0%)	171 (90.0%)	
Type of ACS			
Unstable angina	6 (5.0%)	113 (95.0%)	0.01
Non-ST elevation MI	10 (19.2%)	42 (80.8%)	
ST elevation MI	10 (9.9%)	91 (90.1%)	
Time of presentation of ACS			
1-12 hrs	12 (6.1%)	185 (93.9%)	0.001
>12 hrs	14 (18.7%)	61 (81.3%)	

During the five days of study, there was only one thromboembolic event in the HIT group. The patient developed pulmonary embolism (PE) on Day 3 and did not survive.

## Discussion

Heparin-induced thrombocytopenia (HIT) is by far the most relevant pathological association of heparin therapy encountered by clinicians. It is an immune-mediated phenomenon caused by antibodies directed against complexes of heparin molecules and platelet factor 4 (PF4). HIT is a considerable side effect of heparin therapy in patients with ACS. If the diagnosis is missed, cardiologists, at times, continue or even increase the dose of heparin to counter thrombotic events while the actual management is in stopping heparin and substituting with non-heparin anticoagulation therapy. There was a 9.5% incidence of HIT in this study, which was correlated with the type and delayed presentation of ACS.

Literature has reported a varying incidence of HIT in ACS patients. Where some studies have reported HIT to be as low as only 0.3%, the incidence of HIT has also been reported to be as high as 13% [8-9]. This study also has a comparable outcome. The ischemic necrosis in patients with myocardial dysfunction, which actually leads to acute coronary syndrome, promotes inflammation at the cellular level, which increases platelet clearance by macrophages and acts as the primary stimulus for thrombocytopenia in these patients [9]. Hypotension in these patients may also inhibit platelet production from bone marrow [10]. Decreased platelet count in patients with ACS has been repeatedly reported such as in the CRUSADE trial (Can Rapid Risk Stratification of Unstable Angina Patients Suppress Adverse Outcomes with Early Implementation of the American Cardiology Council/American Heart Association Guidelines), there were 13% NSTEMI patients who developed thrombocytopenia. In the Acute Catheterization and Urgent Intervention Triage strategy trial (ACUITY trial), 6.8% ACS patients developed thrombocytopenia and in Global Registry of Acute Coronary Events (GRACE), there were 1.6% patients with STEMI/NSTEMI who developed thrombocytopenia [8].

Although bleeding is not very common in these patients [11], once HIT has ensued, it has a high association with thromboembolic complications, such as deep venous thrombosis (DVT) and PE, which can be fatal for these patients [12]. Both DVT and PE are more frequently encountered in postoperative patients [13] and the presence of a central venous catheter makes the patient more prone to upper‐extremity venous thrombosis as a complication of HIT. Arterial thrombosis is more common in ACS patients receiving heparin as compared to venous thrombosis [14]. Thrombosis in patients with HIT has a 20%-30% mortality risk [15].

Heparin-induced thrombocytopenia is a critical yet preventable complication of heparin therapy in patients with ACS. This study is primary in contributing to the local data on HIT in ACS. It highlights that HIT is not as uncommon in this patient group. It calls for more clinical investigations to validate these findings, evaluate the predisposing factors, and study the critical consequences of HIT in patients with acute coronary syndrome. However, it has its limitations too. It did not follow the patients for a longer duration to evaluate delayed thromboembolic complications. Immunology was also not done and only thrombocytopenia was used to diagnose HIT. Our hospital is a low-resource one, and lack of funds was our major hindrance. Longitudinal studies are recommended, which can follow the patients to observe for any delayed complications and mortality rate.

## Conclusions

HIT cannot be underestimated as a crucial complication of heparin therapy in patients with acute coronary syndrome. The risk of HIT is more prominent in patients with myocardial infarction and in those who have a duration of symptoms more than12 hours at the time of hospital presentation. Cardiologists and specialists of internal medicine have to take precautions by the serial monitoring of platelet count while administrating heparin therapy in these high-risk patients, to avoid any complications.
